# Supplementary material for: Dynamic collateral sensitivity profiles highlight opportunities and challenges for optimizing antibiotic treatments
Source: PLoS Biol. 2025 Jan 8;23(1):e3002970. doi: 10.1371/journal.pbio.3002970 (PMC11709278; doi:10.1371/journal.pbio.3002970)
Supplement: Attachment — Submitted filename: 2024_finalrevision_response_to_reviewers.pdf [file pbio.3002970.s004.pdf]

Point-by-point responses to reviewers and editors

We thank the reviewers and editors for their continued feedback and discussion. In the text below, we have provided responses to each comment raised (bold text), pointing the reviewers to updates we have made to the manuscript (blue text) in response to their comments/critiques.

Reviewer #1:

Maltas et al submitted a revised version on dynamic collateral sensitivity profiles in an Enterococcus model. I remain very enthusiastic about the manuscript, which the authors again improved substantially. The finding of dynamic collateral effects during the evolution of resistance is an important novel finding, previously not properly documented. The finding is well supported by evolving the model bacterium to high resistance against 5 different antibiotics, combined with comprehensive resistance testing. The initial insights obtained are further corroborated by two types of mathematical models and, in my opinion most important, additional follow-up experiments, including one where the dynamic nature of collateral sensitivity and its potential to constrain bacterial adaptation is evaluated experimentally (see Fig. 4).

I would like to add that in my opinion, the authors have very well addressed the concerns by reviewers. I particularly like the addition of the new model based on dynamic MDP. I also agree with the authors reply on the validity of the findings presented in Figures 1 and 2, and very much welcome the addition of further statistical analyses to support the conclusions drawn. I also agree with the authors that it is very well justified to focus and highlight both global trends as well as individual dynamics in particular populations. In my opinion, the identification of global trends is of particular value, because it highlights prevailing processes that apply across different antibiotics. This manuscript represents a major advance in understanding, especially regarding the here repeatedly shown dynamics of collateral effects. Therefore, this manuscript is very well suited for publication in PLoS Biology.

We thank the reviewer for the kind words and suggestions throughout the submission process. This manuscript has been significantly improved throughout the review process.

I still have a few minor suggestions for improving the manuscript:

1) The authors could emphasize in the introduction and at the beginning of the discussion that Enterococcus faecalis is used as a model to assess the dynamics of collateral effects, thereby making clear that future work in other bacterial taxa is important to further explore the findings made.

We have amended the introduction to include the follow note:

"This work serves to motivate future studies investigating dynamic collateral effects across bacteria species, as well as in cancers, fungi, and viruses."

This is already highlighted in the discussion noted below:

"In addition, we focused on a single bacterial species, and all experiments were performed starting from the ancestral V583 \textit{E. faecalis} strain. It is not clear how

the results might change in a different strain or species, though data from several recent studies suggest the presence of dynamic features in collateral profiles in other species~\cite{imamovic2018Cell, beckley2021identification, roemhild2021mechanisms, batra2021high}."

2) Third last sentence of the abstract: It should be "decreases in isolates.." instead of "..decreases is isolates.."

We thank the reviewer for catching this and have corrected this.

3) Last sentence of abstract: I would mention and thus highlight already in the abstract that the mathematical model is based on dynamic Markov Decision Process.

We agree and have modified to abstract to include this point:

"Finally, we develop a stochastic mathematical model based on a Markov decision process consistent with observed dynamic collateral profiles to show measurements across time are required to optimally constrain antibiotic resistance."

4) Line 31: Gram should be written with a Capital G

We thank the reviewer for catching this and have corrected this.

5) Line 49: I recommend to emphasize that evolution experiments were performed with each of 5 antibiotics listed in table 1. The sentence in lines 47-50 may be mis-read that only 1 antibiotic was used for the evolution experiments.

We agree and have modified to text to include this point:

"To investigate how collateral effects change over time in \textit{E. faecalis}, we exposed four independent evolutionary replicates of strain V583 to escalating concentrations of a single drug over 8 days (approximately 60 generations) via serial-passage laboratory evolution (Fig~\ref{fig1}, Materials and methods). This process was repeated for each drug in Table 1."

6) Line 58: A related point: I would explain that the 20 evolving populations are 4 independent replicate populations evolving in the presence of either of 5 antibiotics, in order to avoid any misunderstanding (such that only a single antibiotic was used for these evolution experiments).

We agree and have modified to text to include this point:

"We quantified resistance and sensitivity by estimating the half-maximum inhibitory dose (IC$_{\text{50}}$) for each strain-antibiotic combination (Materials and Methods). In total, we estimated the IC$_{\text{50}}$ for 400 strain-antibiotic combinations (20 evolving populations made up of 4 independent replicates selected for resistance in the presence of one of the 5 selecting drugs in Table 1, measured against the same 5 antibiotics, at 4 evolutionary time points), each in (technical) replicates of three."

7) Legend to figure 1: I would replace "mutants" with "isolates"

We have made the suggested change.

8) Legend to figure 1: I have the impression that the description of rows and columns is mixed up. The rows show the testing drugs and the columns the selecting drugs. If not, then the labels in the panel should be switched.

We thank the reviewer for catching this error resulting from changes to the old manuscript and have corrected this in the caption.

9) At the beginning of the discussion, I recommend to add that there is a dynamic RELATIVE increase in collateral sensitivity over time, while in comparison with the ancestor cross-resistance dominates.

This is a good point, we have included a brief discussion of this phenomenon at the beginning of the discussion:

"Broadly, we observe an initial strong bias toward collateral resistance, while subsequent evolutionary steps are more balanced between collateral sensitivity and resistance relative to their most recent evolutionary isolate. Often, the initial acquisition of collateral resistance is of sufficient magnitude to counter future relative sensitivity gains, resulting in a less-susceptible population relative to the original ancestor. This conclusion is likely dependent on the length of the overall evolutionary experiment where further evolution may ultimately fully reverse the initial resistance acquisition, however additional studies are required."

10) In the discussion, I would highlight more clearly the very insightful validation experiment shown in figure 4 and also the new model based on dynamic MDP. Both are a particular strength and should thus be more visible.

We agree with the reviewer and have added a section highlighting these components in the discussion:

The time-dependent nature of collateral sensitivity and collateral resistance presents both additional challenges and new opportunities for designing multi-drug therapies to slow resistance. <u>This idea is underscored by our 14 day evolution experiment that highlights the potential transience of collateral sensitivity windows we seek to leverage in treatment (Fig~\ref{fig4}B), in addition to our d-MDP results which reveal just how sensitive optimal treatments are to dynamic underlying collateral profiles (Fig~\ref{fig5}).</u> Full optimization of sequential drug therapies will likely involve not merely static end-point measurements of collateral effects, but a full description of their temporal development.

Reviewer #2:

I was supportive of the prior version and appreciate this version more, especially with the added stochastic control model to evaluate effects of time dependency on resistance constraint. I find the work well written and valuable.

We thank the reviewer for the kind words and suggestions throughout the submission process. This manuscript has been significantly improved throughout the review process.

My broader editorial recommendation is that space and time permitting, the paper would benefit from an additional paragraph in the Discussion that places these studies in context of other work, both on modeling and testing collateral sensitivity evolution in Ef and in biological models with other pathogens or cancers. This would help to connect this study with the field more concretely.

We agree with the reviewer and have added to the discussion the following:

Our results complement several recent studies on collateral effects that suggest sequential therapy may help slow or reverse resistance in bacteria~\cite{batra2021high, barbosa2019Elife, maltas2019PlosB}, fungi~\cite{carolus2024collateral}, and cancers~\cite{zhao2016Cell}. However, sequential therapy has led to mixed results when tested~\cite{abel2014cycling,imamovic2018Cell}. This work suggests that collateral profiles are themselves evolving, providing additional context as to why sequential therapy may not provide the expected treatment benefits. It is not clear how the results might change in a different strain or species, though data from several recent studies suggest the presence of dynamic features in collateral profiles in other species~\cite{imamovic2018Cell, beckley2021identification, roemhild2021mechanisms, batra2021high}.
