# Supplementary material for: Dynamic collateral sensitivity profiles highlight opportunities and challenges for optimizing antibiotic treatments
Source: PLoS Biol. 2025 Jan 8;23(1):e3002970. doi: 10.1371/journal.pbio.3002970 (PMC11709278; doi:10.1371/journal.pbio.3002970)
Supplement: Attachment — Submitted filename: 2024_ResponseToReviewersEditors.docx [file pbio.3002970.s003.docx]

**Point-by-point responses to reviewers and editors**

**We thank the reviewers and editors for their continued feedback and discussion. In the text below, we have provided responses to each comment raised (bold text), pointing the reviewers to updates we have made to the manuscript (blue text) in response to their comments/critiques.**

Editor comments:

Although we do appreciate the points you raise in your letter, we consider that the concerns raised by the referees regarding the main remaining issue after the revision, namely the limited insight gained from the model, are sufficiently important as to prevent publication of your work in PLOS Biology. This point was also highlighted by reviewer #3 saying that the model should be used to generate more quantitative insight. You can also see the comments from the Academic Editor that I have pasted after my signature in this email.

COMMENTS FROM THE ACADEMIC EDITOR:

One reason for sending the manuscript for review was the hope that this model would make a meaningful contribution, which would certainly strengthen this work and make it more interesting to a broader, interdisciplinary audience. This has unfortunately not materialized.

The authors point out that they could easily add a more meaningful model. However, this would be a substantial change, and it is doubtful if it is really so easy to do something convincing along these lines within a few weeks, as they suggest. The idea of this work is very good, but as is, it does not live up to its potential, and I do not think that this can be fixed by a few more improvements.

**We thank the editor and academic editor for their comments surrounding the mathematical model. In order to gain more valuable quantitative insight into the evolutionary dynamics, we have now extended our previous Markov Decision Process (MDP) model to include temporally dynamic collateral profiles which we dub the d-MDP (dynamic Markov Decision Process). The original MDP, first introduced in the original Maltas and Wood 2019 paper (to which the current work is an update), took as input a static collateral sensitivity matrix and assumed the collateral profiles were unchanging. As shown experimentally in this work, that is not always a good assumption. In the revamped model, the d-MDP can take any number of collateral profiles that describe the time-dependent collateral effects. As a result, the d-MDP can solve for the optimal treatment at some time t, given both the current expected collateral evolution, as well as the future possible collateral evolution. This resulted in both a new results section (Dynamic collateral profiles require dynamic treatments to optimally constrain resistance), and a new figure dedicated to these results (Figure 5). For the sake of brevity, we have not included the full additional text below. Instead, we have included several highlights from this work below:**

**“In comparison to the static MDP solutions, we find that d-MDP both better constrains long-term resistance (Figure 5A) and leads to lower total resistance (Figure 5B, ~50% lower total resistance). Crucially, the d-MDP policy incorporates the reward of future collateral profiles allowing for accurate cost-benefit analysis of each action in the context of dynamic collateral profiles.” (Lines 276-280).**

**“We see that over time the policies heavily diverge (day2 optimal vs TD2 is very similar, while day6 and day 8 optimal profiles show large differences in policy with the TD6/TD8 counterparts), reinforcing the importance of measuring dynamic collateral profiles.” (Lines 286-290).**

**“Strikingly, we find that the static MDP policies fail despite creating transient periods of collateral sensitivity. For example, the static MDP policies on Day 6 and Day 8 can result in extremely sensitive states. However, because these sensitivities are not predicted by the static models, these policies fail to exploit them for long-term control. By contrast, the d-MDP policy predictably creates, and optimally leverages, these sensitive states, thereby better constraining resistance.” (Lines 291-297).**

**Put simply, the time-dependent optimal greatly out-performs the time-independent static measures (no matter what time point you measure the static timepoint). Perhaps most interesting from our results is that the static MDP policies do not fail from a lack of sensitive states to treat, but because they fail to predict the sensitive states due to a lack of temporally dynamic collateral profiles. This, more than anything else, highlights the fact that the ability to optimally control resistance populations may require time-dependent measurements of collateral effects.**

Reviewers’ comments:

Reviewer #1: I appreciate the extensive revisions of this updated manuscript. It now more clearly defines the exciting findings that collateral effects shift from resistance in early phases of drug adaptation to a mix of sensitivity and resistance in the latter stages, and also points to likely population- and molecular-genetic causes of these patterns. I think it's a strong next step from this team's work.

**We would like to thank the reviewer for their thoughtful comments throughout the review process that improved this work, especially in its communication and presentation. We are happy the reviewer found our past revisions satisfactory.**

Reviewer #2: The manuscript is substantially improved, and the most problematic parts have been removed. It still has problems.

**We are happy the reviewer notes that the manuscript is substantially improved. We believe we have now addressed these problems, some of which may have resulted from a misunderstanding that we hope to clarify.**

While multiple colonies from the evolution experiments have now been examined in figure 3, Figures 1 and 2, and the poorly-supported conclusions from single replicates in those experiments remain essentially unchanged.

**The analysis leading to the claims in Figures 1 and 2 were substantially changed in the previous submission – thanks in great part to this reviewer’s original comments – and we believe the paper is much improved because of it. Please note that no claims are related to, or depend on, this data being from single isolates (and we note in passing that they do all involve multiple technical replicates, though that is clearly not the reviewer’s point).**

**Specifically, all claims related to Figures 1 and 2 are now accompanied by rigorous statistical tests, which were admittedly missing from the original draft – in part because the empirical data was quite strong, even in the absence of formal tests. Nevertheless, the work is undoubtedly stronger with the statistical tests, and they were needed, as the reviewer noted. Here’s a brief description of each figure’s purpose and the associated claims:**

**Figure 1 includes a schematic and a simple visualization of experimental data: the relative IC50 from individual colonies sampled over time from different drug-adapted populations. We make only descriptive statements about the data and we do not intend to make any strong claims here – the figure is largely for pedagogical purposes and to show the reader the experimental set-up.**

**Figure 2, on the other hand, demonstrates one of the primary claims of the work: *At a global level – when results from all drugs are combined – collateral resistance dominates during early phases of adaptation, when resistance to the selection drug is lower, while collateral sensitivity becomes increasingly likely with further selection.* This finding is supported in two ways:**

1. **We show that the mean instantaneous collateral effects decrease (i.e. collateral effects become more sensitive) as resistance to selecting drug is increased (panel 2C, left).**
2. **We show that the probability (frequency) of collateral sensitivity increases with resistance to the selecting drug (panel 2C, right).**

**These plots are supported by rigorous statistical tests (e.g. logistic regressions) and also appear when we look at the data in different ways. For example, the **cumulative** collateral effects also decrease as resistance to the selecting drug increases (Figure S1), and the probability of collateral resistance decreases with resistance to selecting drug (Figure S2). In addition, there are clear trends in the distribution of instantaneous collateral effects over time (days), as shown in colormaps in Figure 2. Again, all of these claims are related to global trends in the CS data across drugs, not to single isolates.**

**With the exception of colormaps in Figure 2, these analyses represented new additions** **included in the previous revision, but not the original draft. In our view, these additions provide substantial evidence to support the claims. We’ve attempted to be clear and transparent about these claims, and we are open to performing additional statistical tests or analyses – and even more experiments if warranted – but at this stage it is not clear to us why the reviewer believes these methods are insufficient to support the claims.**

The paper is stronger for lack of the details of the "toy model", though now that section does not seem to be doing anything and the paper is likely strengthened by removing it entirely.

**We have now added substantial additions to the modeling portion of the manuscript by extending the Markov Decision Process (MDP) model used in our original 2019 PLoS Bio paper to include temporally dynamic collateral profiles. We detail these new additions in several responses below. In addition to the MDP model, we have still included some analysis with the original toy model, whose purpose is to 1) illustrate the combinatorial complexity potentially associated with dynamic collateral sensitive profiles – even in the case of a small set of drugs – and 2) to identify drug pairs where such dynamic profiles significantly impact inhibitory effects of a second drug – a qualitative prediction that guided the design of the following experiment in the manuscript.**

The new Figure 3C is also misleading as the continuous lines imply that the data points are descendents of one another and not both shallow samples from populations that descended from one another.

**We are puzzled by this comment. We certainly did not intend to be misleading, and we have tried to clarify the figure caption and text. As we explain there, each panel in Figure 3C shows the frequency of isolates with CS or CR drawn from a single population at four different time points. The population at time point t+1 does, of course, descend from the population at the previous time point t. But by nature, frequency is a measure of a population effect – a macroscopic observable estimated from sampling in a population – and therefore it is not clear in what other ways the frequency from one time point could be a descendent from a previous time point. Perhaps the reviewer thought we were plotting IC50 over time and claiming it represented the change in resistance in a single lineage? That is certainly not what we intended. We do note, however, that in Figure 2, when analyzing the global trends across all drugs, we plotted the mean collateral effects as a function of resistance to the selecting drug, and the continuous line is clearly labeled as a moving average.**

Overall, the revised manuscript and data seem to be saying "a lot can happen in both directions and this makes it harder or easier to use antibiotic sequences" -- which seems to be saying two things that are contradictory simultaneously. It remains unclear what the well-supported conclusion of the paper is beyond "it's all stochastic"

**We do not agree with this characterization of our claims and results, but the reviewer’s perspective suggests we were not sufficiently clear in the previous drafts. Indeed, we do highlight and acknowledge that stochastic effects play a major role in evolution, especially at the level of individual lineages. However, we make two distinct claims related to collateral trends – one at the global level (across all drugs) and one at the single population level – and those statements are in no way contradictory.**

**First, the global trend: when we combine results across all drugs, we find that early stages of adaptation are associated with increased frequency of collateral resistance and a larger average collateral effect, while later stages are associated with increased frequency of collateral sensitivity and a smaller (more negative) average collateral effect (Figure 2C, Figure S2). These trends are supported by logistic regression and appear when the data is viewed in different ways – for example, similar trends arise when we measure the frequency of isolates exhibiting instantaneous collateral sensitivity over real time (top panels in Figure 2). In our view, these are important global trends, even if they do not predict what may happen in any individual population or selecting drug.**

**Given this global trend, we then asked (thanks to the interesting suggestion of the reviewer at the first stage of review) whether individual populations truly show time-dependent changes in collateral effects. To investigate whether temporal trends might occur in individual populations – that is, whether the distribution of collateral profiles changes over time, not merely due to sample noise at different time points – we isolated and phenotyped 12 colonies from three populations at 4 time points. (We note that these experiments comprise over 500 new dose-response curves – more than 5000 individual measurements – that were not included in the original manuscript but represent significant additional work). We found that, indeed, the distribution of collateral effects changes over time. In 2/3 populations, the trends we observe globally (decreasing collateral resistance / increasing collateral sensitivity over time) are also observed in single populations, while the third population exhibits the opposite trend. These results indicate that the distribution of collateral effects is dynamic, even in individual populations, though we make no claim about the evolutionary trend of any single population or even the distribution of trends in an ensemble of populations. These results do not contradict the global trend, but instead they demonstrate dynamic but idiosyncratic behavior of CS profiles in individual populations, a finding that was missing from our original manuscript.**

**In addition to clarifying the points above, in the newest draft we thoroughly investigate the impacts of these trends on population control using a stochastic control model. To do so, we extended the stochastic mathematical model used in our previous manuscript (Maltas and Wood. PLoS Biology (2019)) to account for temporally dynamic collateral profiles (the previous model was limited to only a single static collateral profile measurement/timepoint). The result of this is a large addition to the results section (Dynamic collateral profiles require dynamic treatments to optimally constrain resistance), and a new figure dedicated to these results (Figure 5). For the sake of brevity, we do not include the whole section here, but offer a few insights:**

**“In comparison to the static MDP solutions, we find that the time-dependent control strategy both better constrains long-term resistance (Figure 5A) and leads to lower total resistance (Figure 5B, ~50% lower total resistance). Crucially, the d-MDP (time-dependent) policy incorporates the reward of future collateral profiles allowing for accurate cost-benefit analysis of each action in the context of dynamic collateral profiles.” (Lines 276-280).**

**“Strikingly, we find that the static MDP policies fail despite creating transient periods of collateral sensitivity. For example, the static MDP policies on Day 6 and Day 8 can result in extremely sensitive states. However, because these sensitivities are not predicted by the static models, these policies fail to exploit them for long-term control. By contrast, the d-MDP policy predictably creates, and optimally leverages, these sensitive states, thereby better constraining resistance.” (Lines 291-297).**

**We believe this analysis highlights the feasibility of population control—despite the inherent stochasticity of the system—while also underscoring the limitations of static strategies that do not incorporate dynamic collateral effects.**
